# Identifying predictors of clinical outcomes using the projection-predictive feature selection—a proof of concept on the example of Crohn’s disease

**DOI:** 10.3389/fped.2023.1170563

**Published:** 2023-07-28

**Authors:** Elisa Wirthgen, Frank Weber, Laura Kubickova-Weber, Benjamin Schiller, Sarah Schiller, Michael Radke, Jan Däbritz

**Affiliations:** ^1^ Department of Pediatrics, Rostock University Medical Center, Rostock, Germany; ^2^ Institute for Biostatistics and Informatics in Medicine and Ageing Research, Rostock University Medical Center, Rostock, Germany; ^3^ Medical School, University of Rostock, Rostock, Germany; ^4^ Department of Pediatrics, Pediatric Gastroenterology, Rostock University Medical Center, Rostock, Germany; ^5^ Department of Pediatrics, Greifswald University Medical Center, Greifswald, Germany

**Keywords:** inflammatory bowel disease, endoscopy, calprotectin, C-reactive protein, monitoring, Bayesian, ordinal regression model, Shiny application

## Abstract

**Objectives:**

Several clinical disease activity indices (DAIs) have been developed to noninvasively assess mucosal healing in pediatric Crohn’s disease (CD). However, their clinical application can be complex. Therefore, we present a new way to identify the most informative biomarkers for mucosal inflammation from current markers in use and, based on this, how to obtain an easy-to-use DAI for clinical practice. A further aim of our proof-of-concept study is to demonstrate how the performance of such a new DAI can be compared to that of existing DAIs.

**Methods:**

The data of two independent study cohorts, with 167 visits from 109 children and adolescents with CD, were evaluated retrospectively. A variable selection based on a Bayesian ordinal regression model was applied to select clinical or standard laboratory parameters as predictors, using an endoscopic outcome. The predictive performance of the resulting model was compared to that of existing pediatric DAIs.

**Results:**

With our proof-of-concept dataset, the resulting model included C-reactive protein (CRP) and fecal calprotectin (FC) as predictors. In general, our model performed better than the existing DAIs. To show how our Bayesian approach can be applied in practice, we developed a web application for predicting disease activity for a new CD patient or visit.

**Conclusions:**

Our work serves as a proof-of-concept, showing that the statistical methods used here can identify biomarkers relevant for the prediction of a clinical outcome. In our case, a small number of biomarkers is sufficient, which, together with the web interface, facilitates the clinical application. However, the retrospective nature of our study, the rather small amount of data, and the lack of an external validation cohort do not allow us to consider our results as the establishment of a novel DAI for pediatric CD. This needs to be done with the help of a prospective study with more data and an external validation cohort in the future.

## 1. Introduction

Mucosal healing, reflected by endoscopic remission, has become a significant endpoint of Crohn’s disease (CD) therapy and is associated with long-term clinical remission (1). Studies investigating mucosal healing are often based on endoscopic measures of disease activity and treatment response (2). Endoscopic mucosal healing is defined as the resolution of visible inflammation and ulceration during endoscopy. The use of endoscopic indices remains the gold standard for the assessment of inflammatory activity. However, there are several limitations of endoscopic procedures, including invasiveness, risks due to sedation, and high financial costs. Especially for pediatric patients, proxies of endoscopic mucosal healing are needed since an endoscopy in children usually requires hospitalization for a successful bowel cleansing/preparation and also sedation or anesthesia, which increases the risk of complications (3). Therefore, several clinical disease activity indices (DAIs) were developed using noninvasive clinical and standard laboratory parameters for monitoring the severity of CD-associated intestinal inflammation in clinical practice. For example, the Pediatric Crohn’s Disease Activity Index (PCDAI) (4) is determined regularly in the CEDATA-GPGE registry including data of 6,233 pediatric patients with inflammatory bowel disease (IBD) in 2022 (5). However, the PCDAI, developed in 1991, has not been validated against established endoscopic disease activity scores and includes subjective variables which may potentially compromise its predictive performance. Furthermore, there are indications that the PCDAI is a poor marker of endoscopic disease severity at diagnosis and a poor predictor of endoscopic treatment success (6). Additionally to the PCDAI, five other DAIs for pediatric CD have been developed (7–11), underlining the need for valid noninvasive scores. Investigated parameters involve medical history, physical examination, and laboratory parameters (Table 1). However, the clinical application of those existing DAIs can be complex, either because many parameters need to be collected or because of the complicated and time-consuming calculation.

**Table 1:** Overview of different indices used to assess the clinical disease activity in pediatric Crohn’s disease.

Index	PCDAI	abbrPCDAI	modPCDAI	shPCDAI	wPCDAI	MINI
Year/Reference	1991 (4)	2004 (7)	2010 (8)	2011 (9)	2011 (10)	2019 (11)
Medical history						
Abdominal pain	x	x	x	x	x	
Number of stools per day	x	x	x	x	x	x
General well-being	x	x	x	x	x	
Physical examination						
Body weight	x	x	x	x	x	
Linear growth	x		x			
Abdominal findings^a^	x	x	x	x		
Perianal disease	x	x	x		x	
Extra-intestinal manifestations	x	x	x	x		
Laboratory findings						
Hematocrit	x^b^		x^b^			
Erythrocyte sedimentation rate	x		x		x	x^c^
Albumin	x		x		x	
C-reactive protein			x			x^c^
Fecal calprotectin						x
Scoring						
Range	0–100	Not determined	0–115	0–90	0–125	−3–25
Remission	≤10	Not determined	<7.5	<15	<12.5	<8
Mild	11–30	Not determined	7.5–10	15–30	12.5–40	8–11
Moderate to severe	≥31	Not determined	≥12.5^d^	≥30	>40^d^	>11

^a^ No tenderness, no mass; tenderness, or mass without tenderness; tenderness, involuntary guarding, definite mass.

^b^ Scoring is dependent on sex and age.

^c^ Either C-reactive protein or erythrocyte sedimentation rate could be used for calculation, the use of both parameters is preferred.

^d^ Additional cut-off for “severe”: >17.5 (modPCDAI), >57.5 (wPCDAI).

PCDAI, Pediatric Crohn’s Disease Activity Index; abbr, abbreviated; mod, modified; sh, short; w, weighted; MINI, Mucosal Inflammation Noninvasive Index.

Therefore, our proof-of-concept study serves to demonstrate how the most informative biomarkers for mucosal inflammation can be identified from current markers in use and how these results can be used for obtaining an easy-to-use DAI for clinical practice. In total, we included 24 parameters, so-called candidate predictors, to examine their potential to reflect the observed endoscopic inflammation. The candidate predictors included noninvasive parameters, assessed in the medical examination, and laboratory parameters, including common clinical serum parameters such as C-reactive protein (CRP). In terms of the statistical methodology, we applied variable selection based on a Bayesian ordinal regression model with endoscopic inflammation as the outcome. One advantage of this method is that there is no need for pre-selection or weighting of parameters to select the most informative parameters for the chosen outcome.

Our proof-of-concept study also shows how the predictive performance of our model can be compared to that of previously described DAIs. Finally, a Shiny (12) web application was developed to demonstrate how such a biomarker-based DAI could be calculated easily in practice.

## 2. Material and methods

### 2.1. Patients

A total of 167 visits from 109 children and adolescents with an established diagnosis of CD were reviewed retrospectively. These data were derived from two German pediatric IBD centers at the University Medical Center in Rostock (Department of Pediatrics) and at the Klinikum Westbrandenburg (Department of Pediatrics) in Potsdam. General patients’ characteristics are summarized in Table 2. We included visits from pediatric CD patients (≤18 years) in whom the terminal ileum was reached during endoscopy complemented by an esophagogastroduodenoscopy. Visits where patients were suspected of acute infections were not included in our study. The presence of an acute infection has been ruled out by medical history within 14 days before the visit and a complete physical examination by the attending pediatrician. Furthermore, visits where patients had fever (>38°C) were excluded from endoscopic assessment and, therefore, not included in the study. In addition, the diagnostic evidence of pathogenic bacteria (*Campylobacter*/*Salmonella*/*Shigella*/*Vibrio*/*Aeromonas* spec., *Yersinia enterocolitica*, *Clostridium difficile*) or viruses (norovirus, rotavirus, adenovirus, astrovirus, sapovirus) in the patient’s stool was a contraindication for performing an endoscopy. Fecal calprotectin (FC) was recorded ≤30 / ≥0 days before endoscopy (b.e.) (50%: 0–3 days, 13%: 4–10 days, 37%: 11–30 days b.e.). Serum markers were recorded at ≤14 / ≥0 days b.e. (84%: 0–1 day, 12%: 2–3 days, 3%: 5–7 days, 1%: 13 days b.e.). The conduction of this study was approved by the ethics committees of both participating centers [registration numbers A 2020-0161 (Rostock) and AS 73(bB)/2020 (Potsdam)].

**Table 2:** Characteristics of the pediatric Crohn’s disease study cohort.

General characteristics	Patients (total: 109)
Male, *N* (%)	74 (67.9)
Female, *N* (%)	35 (32.1)
Age, years	Q0: 2.5, Q25: 10.9, Q50: 12.4, Q75: 14.5, Q100: 17.7
Body weight, kg	Q0: 13.4, Q25: 29.2, Q50: 40.2, Q75: 51.9, Q100: 107.0
Body height, cm	Q0: 96.0, Q25: 140.8, Q50: 154.0, Q75: 166.2, Q100: 190.0
Endoscopic score, *N* (%)	Visits (total: 167)
Remission	13 (7.8)
Mild	8 (4.8)
Moderate	62 (37.1)
Severe	84 (50.3)

Age, body weight, and body height refer to the first visit of each patient.

Continuous variables are presented as minimum (Q0), 1st quartile (Q25), median (Q50), 3rd quartile (Q75), and maximum (Q100).

### 2.2. Assessment of endoscopic inflammation

Intestinal inflammation of all study participants was routinely assessed endoscopically in both participating IBD centers. Physicians’ findings regarding endoscopic inflammation were used retrospectively to assign patients to 4 grades of inflammation (remission, mild, moderate, and severe). Esophagogastroduodenoscopy and an ileocolonoscopy were performed by trained pediatric gastroenterologists examining pre-defined bowel segments. Thereby, endoscopic inflammation was defined as inactive disease, mild disease (erythema, decreased vascular pattern, mild friability), moderate disease (marked erythema, absent vascular pattern, friability, erosions, small aphthous lesions), or severe disease (spontaneous bleeding, ulceration, extensive “snail track ulcerations”). In doing so, we used the highest endoscopically determined inflammation as a reference for the severity of the inflammation (1–4), irrespective of the location of the inflammation.

### 2.3. Candidate predictors for the statistical model

**Figure 1:**
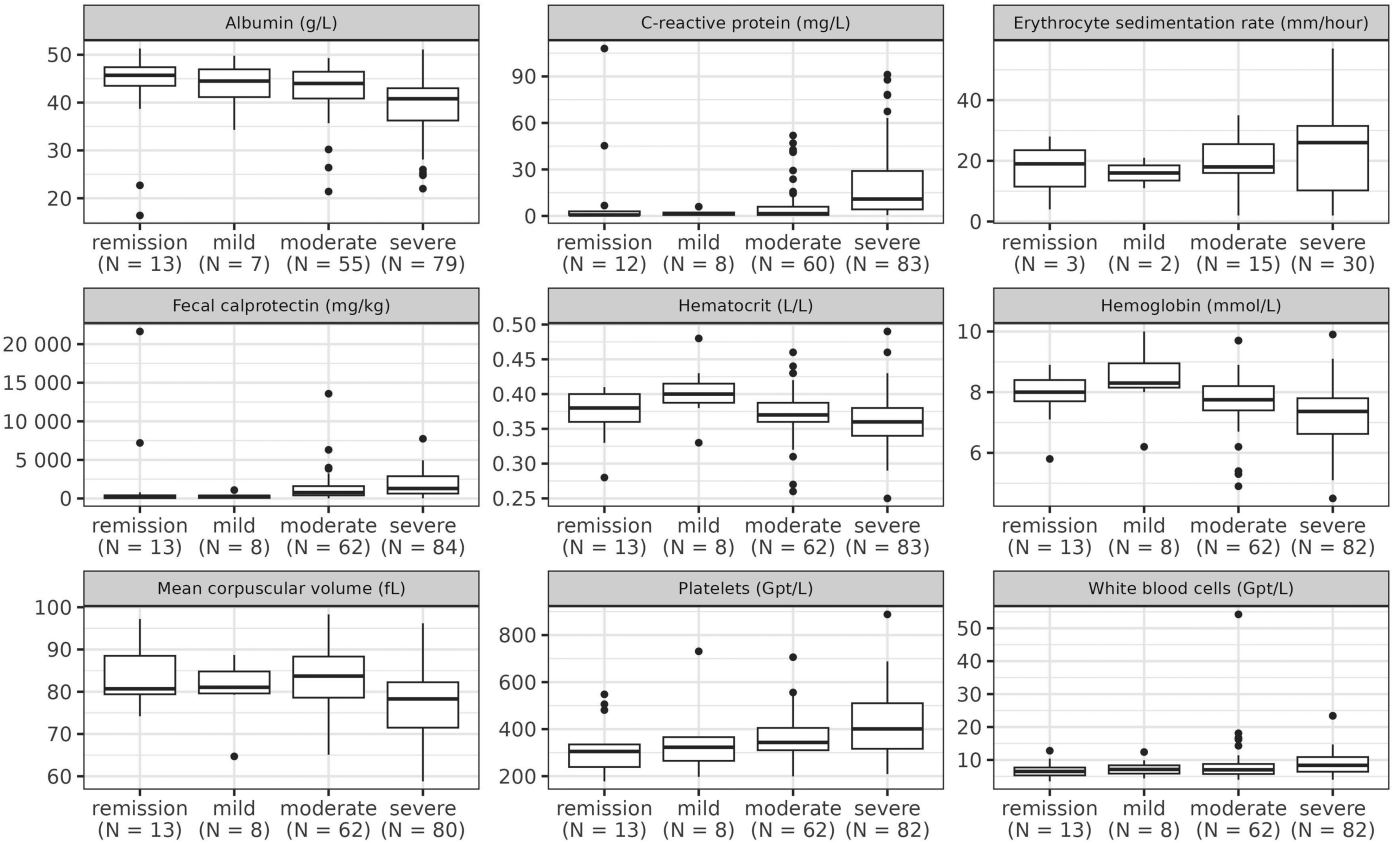
Distribution of laboratory parameters across the categories of the endoscopic score. The boxes of the boxplots consist of lower hinge, median, and upper hinge.

Noninvasive clinical parameters assessed in current DAIs (Table 1) and standard laboratory parameters were considered as candidate predictors in the Bayesian regression model. The noninvasive parameters included age, weight gain, well-being, limitation of daily activities, number of bowel movements and stool consistency, the occurrence of visible blood in stool, abdominal pain (overall as well as at night), abdominal pain on palpation, abdominal resistance, perianal eczema, anal findings, and the presence of extra-intestinal manifestations. The laboratory findings included serum concentrations of albumin, C-reactive protein (CRP), hematocrit, hemoglobin, mean corpuscular volume (MCV), platelets, leukocytes, and FC. The empirical distributions of the laboratory parameters across the endoscopic score categories are presented in Figure 1. That figure also visualizes the erythrocyte sedimentation rate (ESR), which had to be excluded as a candidate predictor due to a high number of missing values. Details concerning categorical predictors are given in Table 3. Another—rather technical—candidate predictor was the IBD center. Furthermore, we considered a term adjusting for the dependence of multiple visits per patient as a candidate predictor (see the Supplementary Appendix).

**Table 3:** Categorical candidate predictors. The frequencies refer to the number of visits (total: 167).

Name	Description
Abdominal pain	88 (53%) “Yes”, 76 (46%) “No”, 3 (2%) MVs
Abdominal pain (night)	3 (2%) “Yes”, 162 (96%) “No”, 3 (2%) MVs
Abdominal finding	123 (74%) “Without pathological findings”, 42 (25%) “With pathological findings”, 2 (1%) MVs
Abdominal finding—pressure pain	130 (78%) “Yes”, 35 (21%) “No”, 2 (1%) MVs
Abdominal finding—resistance	21 (13%) “Yes”, 144 (86%) “No”, 2 (1%) MVs
Anal finding^a^	16 (10%) “Yes”, 149 (89%) “No”, 2 (1%) MVs
Appetite^b^	76 (46%) “Good”, 19 (11%) “Reduced”, 15 (9%) “Poor”, 57 (34%) MVs
Activity limitation	70 (42%) “Yes”, 85 (51%) “No”, 12 (7%) MVs
IBD clinic	58 (35%) “IBD center A”, 109 (65%) “IBD center B"
Condition^c^	106 (63%) “Very good, good”, 58 (35%) “Reduced, poor, very poor”, 3 (2%) MVs
Extraintestinal manifestation^d^	15 (9%) “Yes”, 150 (90%) “No”, 2 (1%) MVs
Height gain^b,e^	159 (95%) “Yes”, 4 (2%) “No”, 4 (2%) MVs
Perianal eczema^b^	4 (2%) “Yes”, 161 (96%) “No”, 2 (2%) MVs
Stool blood	37 (22%) “Yes”, 122 (73%) “No”, 8 (5%) MVs
Stool consistency	63 (38%) “Formed”, 95 (57%) “Semi-formed or liquid”, 9 (5%) MVs
Stool quantity	127 (76%) “≤3 stools per 24 h”, 25 (15%) “>3 stools per 24 h”, 15 (9%) MVs
Weight gain^e^	103 (62%) “Weight gain, voluntary stable weight, voluntary weight loss”, 62 (37%) “Involuntary stable weight, involuntary weight loss”, 2 (1%) MVs

^a^ “Yes” is defined as at least one of the following: rhagade, fissure, indolent/active fistula, abscess, multiple/inflamed tag, abscess.

^b^ Excluded due to a high number of missing values (MVs)/rare categories.

^c^ General well-being and limitations in daily activities.

^d^ “Yes” is defined as at least one of the following: fever ≥38.5°C for more than three days, arthritis, uveitis, erythema nodosum, pyoderma gangrenosum.

^e^ approx. 3–6 months before clinic visits.

### 2.4. Statistics

The concept of our statistical analysis was to fit a Bayesian ordinal regression model (the “reference model”) to the endoscopic inflammation as outcome and to perform predictor selection (also known as “variable” or “feature” selection). In the following, we only give a short outline of our statistical approach. Further details may be found in the Supplementary Appendix. There, we also explain the rationale behind our approach.

For fitting the reference model, we used the R (13) package brms (14–17) which relies on the Stan (18) software in the background. The ordinal endoscopic score constituted the outcome for which we used the “cumulative” distributional family with the “logit” link function. As predictors with population-level (also known as “fixed”) effects, we used the IBD center, the noninvasive parameters, and the laboratory findings described in the previous section. Some laboratory parameters (CRP, platelets, leukocytes, and FC) had an extremely right-skewed distribution, which is why we log-transformed them prior to any modeling steps. Due to the possibility of multiple visits per patient, we included group-level (also known as “random”) effects for the patient identifiers (IDs). These account for the dependence of the visits coming from the same patient. Since a regression model requires non-missing values for all predictors as well as for the outcome, we excluded visits with missing values from the initial dataset with 167 visits, giving a reduced dataset of 131 visits. Afterwards, we used a regularized horseshoe (RH) prior (19) for the population-level regression coefficients and brms’s default priors for the remaining parameters. We centered the predictor variables to mean zero in order to simplify interpretation. As recommended for the RH prior (19), we also scaled the (centered) predictor variables to unit standard deviation. After fitting the reference model, we checked the convergence of the Markov chains using well-established diagnostics and their recommended thresholds (20–22).

The predictor selection method used here is the projection-predictive feature selection (PPFS) implemented in the R package projpred (23–25). Briefly, the PPFS yields a model consisting of the smallest subset of predictors which still achieves a predictive performance as close as possible to the reference model’s predictive performance. For the projections associated with the PPFS, we applied projpred’s latent projection (26) (see the Supplementary Appendix for details).

We calculated the PCDAI (4), abbreviated PCDAI (abbrPCDAI) (7), modified PCDAI (modPCDAI) (8), short PCDAI (shPCDAI) (9), weighted PCDAI (wPCDAI) (10), and the Mucosal Inflammation Noninvasive Index (MINI) (11) for all visits from our dataset (at least where possible, given missing values) and calculated their predictive probabilities for the observed outcome categories by using them as predictors in their own (separate) Bayesian ordinal regression models. These predictive probabilities of the existing DAIs were compared to those of our selected model from the PPFS.

## 3. Results

### 3.1. Predictor selection

**Figure 2:**
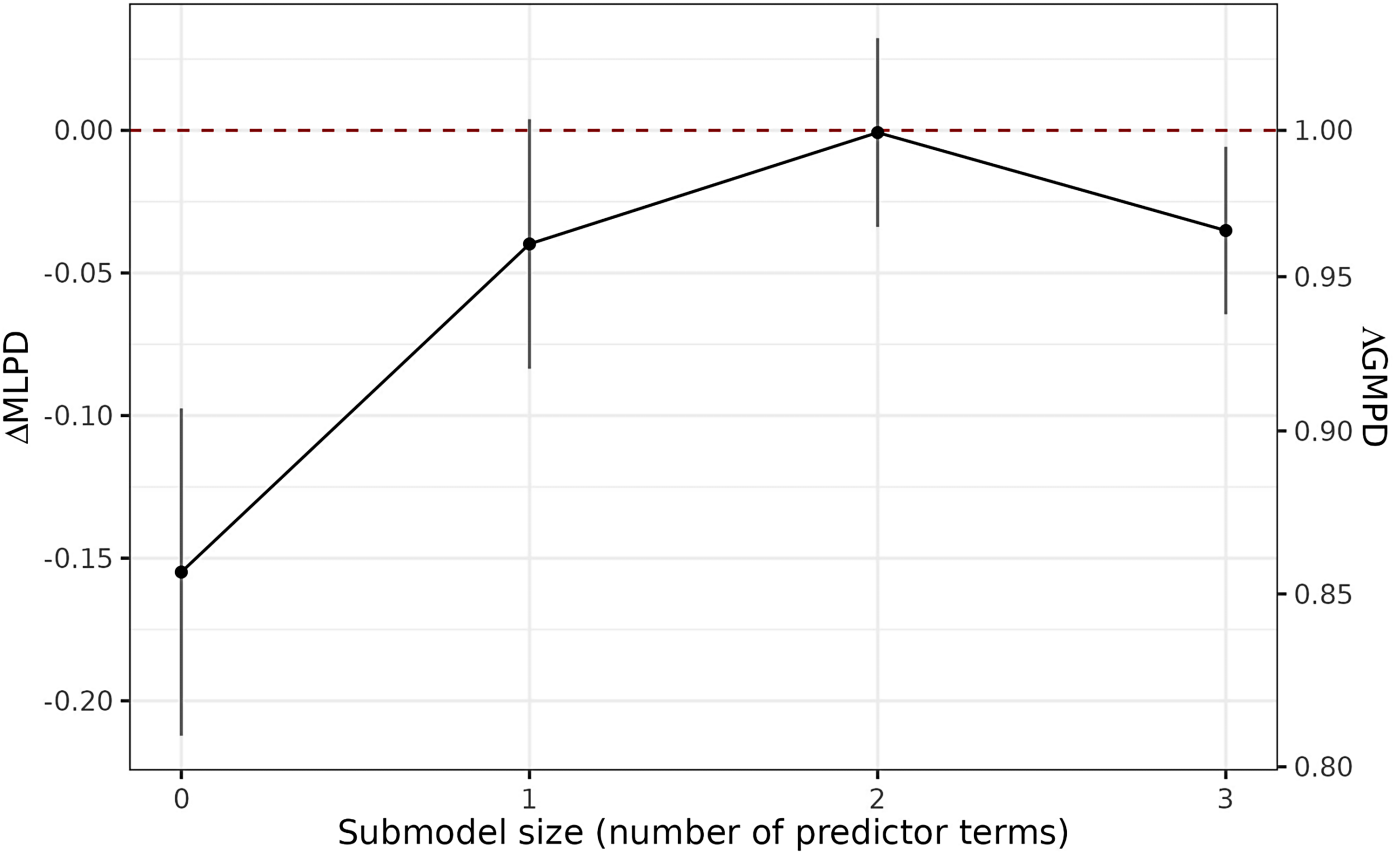
Model size selection plot from the PPFS. This plot is based on the mean log predictive density (MLPD) as predictive performance measure on the left *y*-axis which, when exponentiated to the base of the natural logarithm, gives the geometric mean predictive density (GMPD) on the right *y*-axis. Here, the GMPD is the geometric mean of the predictive probabilities at the observed outcome categories and thus restricted to the interval [0, 1]. The higher the MLPD or the GMPD, the better the predictive performance. The x-axis shows the number of predictors during the forward search. The dashed red line indicates the reference model’s predictive performance, which is here by definition 0 (on the left *y*-axis) and 1 (on the right *y*-axis) since on the left *y*-axis, the plot visualizes ΔMLPD, defined as the submodel MLPD minus the reference model MLPD (and on the right y-axis, the exponentiation gives ΛGMPD, the ratio of the submodel GMPD to the reference model GMPD). The uncertainty bars here indicate ±1 standard error of the *Δ*MLPD estimator.

The model size selection plot for the PPFS indicates that two predictors are sufficient to obtain a predictive performance close to that of the reference model (Figure 2). According to the PPFS’s full-data predictor ranking, these two predictors are CRP and FC (Table 4). Additional predictors, such as the presence of anal findings, did not improve the model’s predictive performance further (Figure 2). Thus, in the final projection (see the Supplementary Appendix), we project the reference model onto the submodel consisting of the predictors CRP and FC, yielding our selected endoscopic submodel (SESM).

**Table 4:** Predictor ranking for endoscopic inflammation based on the projection-predictive feature selection (PPFS; see the Supplementary Appendix for details).

Submodel size	Log CRP	Log FC	Anal finding: “Yes” vs. “No"
1	0.96	0	0.04
2	0.04	0.92	0
3	0	0.08	0.48

The order of the last 3 column names follows the PPFS’s full-data predictor ranking. For each submodel size *m* (first column), the values from the last 3 columns give the proportions of cross-validation (CV) folds which have the predictor from the respective column at position *m* of their forward search’s predictor ranking (there is one forward search per CV fold). Note that the proportions don’t need to sum to 1 (neither row-wise nor column-wise) because the forward search was terminated at submodel size 3 (which is less than the number of predictor terms in the reference model). Apart from the patient ID (not shown here), all predictors were standardized (centered and scaled) prior to modeling (see the Supplementary Appendix). Here, “log” is the natural logarithm. See Table 3 for details on the categorical predictor “anal finding”.

CRP, C-reactive protein; FC, fecal calprotectin.

### 3.2. Selected predictors CRP and FC

**Figure 3:**
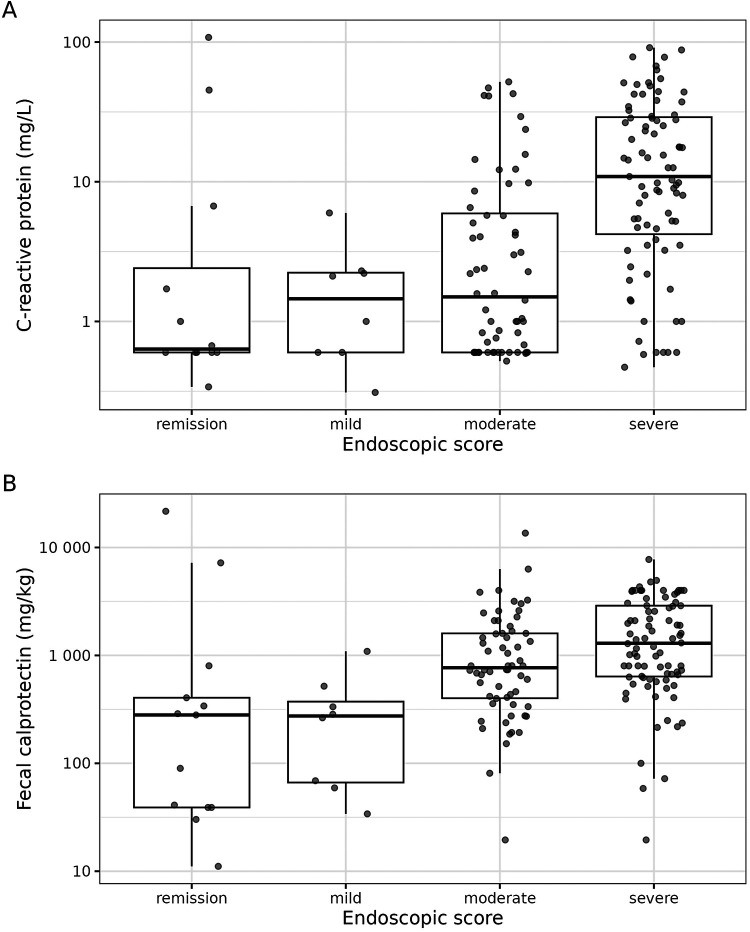
Distribution of C-reactive protein (**A**) and fecal calprotectin (**B**) across the endoscopic score categories. The boxes of the boxplots consist of lower hinge, median, and upper hinge. The *y*-axes are log-scaled.

**Figure 4:**
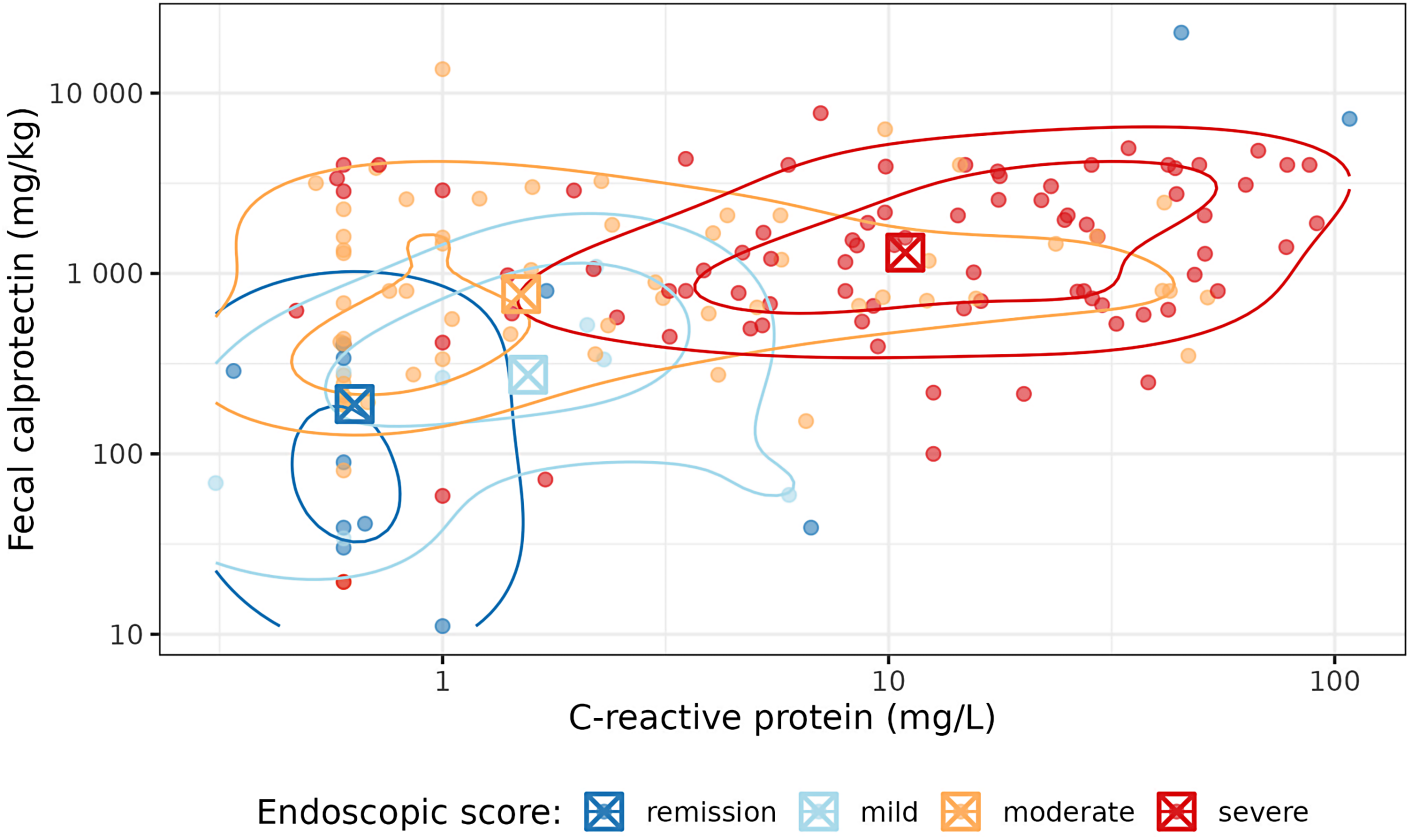
Joint distribution of C-reactive protein and fecal calprotectin, together with their association with the endoscopic score. The contour lines illustrate two-dimensional kernel density estimates. The boxed crosses indicate the category-wise medians of C-reactive protein and fecal calprotectin. Both axes are log-scaled.

**Figure 5:**
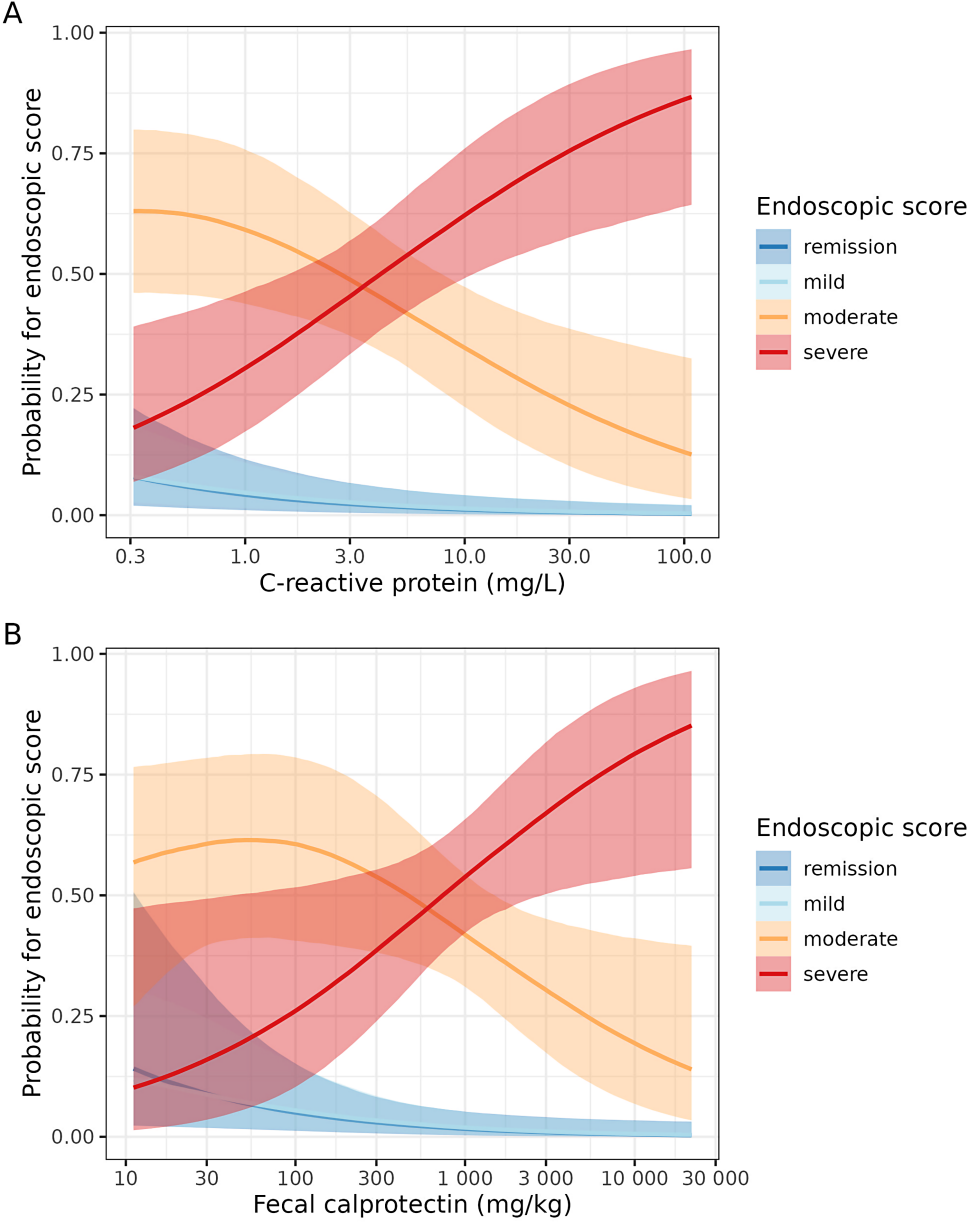
Estimated projected effect of C-reactive protein (**A**) and fecal calprotectin (**B**) on the endoscopic score (conditional-effects plots from the selected endoscopic submodel, SESM). Note that these plots should not be interpreted as showing the isolated effects of C-reactive protein and fecal calprotectin since they are based on the *projected* posterior (see the Supplementary Appendix). Furthermore, these plots condition on the mean (standardized and log-transformed) fecal calprotectin and C-reactive protein [for (**A,B**), respectively]. The semi-transparent bands indicate 95% uncertainty (projected posterior) intervals. The *x*-axes are log-scaled.

The empirical distributions of CRP and FC across the endoscopic score categories are presented in Figures 3A,B, respectively. These plots show that (in general) the endoscopic severity increases with the concentrations of CRP and FC. However, especially the FC values in the group of endoscopic remission reveal a high variance. The joint distribution of CRP and FC, together with their association with the endoscopic score, is presented in Figure 4. This plot illustrates again the association of CRP and FC with the endoscopic score, although the variability of CRP and FC within the endoscopic score categories is considerable, leading to some overlap of the “clusters” formed by the differently colored score categories. The relationship of CRP and FC with the endoscopic score is also reflected in Figures 5A,B (there not from a descriptive point of view, but from a modeling perspective—using the SESM).

### 3.3. Comparison to existing pediatric CD activity indices

**Figure 6:**
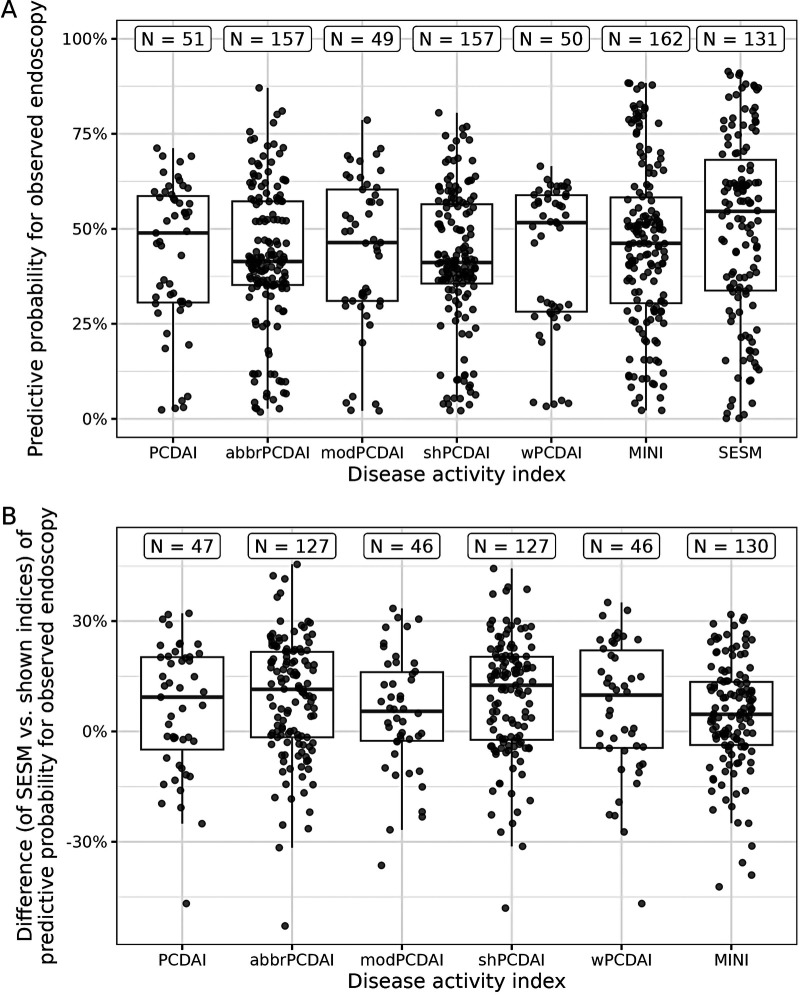
Comparison of the selected endoscopic submodel (SESM) vs. existing disease activity indices (DAIs) for pediatric Crohn’s disease. (**A**) Predictive probability for the observed endoscopic score of each existing DAI and the SESM. (**B**) The predictive probability of the SESM minus the predictive probability of each existing DAI. The boxes of the boxplots consist of lower hinge, median, and upper hinge. At the top, “N” indicates the number of visits in the dataset used for the corresponding boxplot. PCDAI, Pediatric Crohn’s Disease Activity Index; abbr, abbreviated; mod, modified; sh, short; w, weighted; MINI, Mucosal Inflammation Noninvasive Index.

The comparison of the SESM to the existing DAIs is illustrated in Figure 6. Figure 6A shows the predictive probabilities for the observed endoscopic inflammation categories, and Figure 6B shows the corresponding differences by which the SESM can be compared to the existing DAIs directly. The fact that most of the differences in Figure 6B are positive suggests a superiority of the SESM compared to the existing DAIs. Note that all this is based on our proof-of-concept dataset and hence should not be over-interpreted, also because the DAIs are based on different numbers of visits (as indicated by “N” in Figure 6; see also the Supplementary Appendix) due to missing values in their corresponding predictors (we conducted separate complete-case analyses).

### 3.4. Application of the SESM

In contrast to the existing DAIs, the SESM is not intended to yield a single value on a scale of, e.g., 0–100. To show how the SESM can be applied easily nonetheless, we have created a Shiny (12) web application (accessible at https://umrukj.shinyapps.io/sesm/) where the user enters values for CRP and FC and obtains the predictive probabilities for each of the four endoscopic score categories (remission, mild, moderate, severe). We calculated these predictive probabilities for preselected CRP and FC values (Table 5). For example, measured concentrations of 1 mg/kg FC and 1 mg/L CRP lead to a probability of 56% for endoscopic remission, followed by 14%, 26%, and 4% for mild, moderate, or severe endoscopic inflammation, respectively. An increased concentration of 500 mg/kg FC while keeping a CRP concentration of 1 mg/L results in an increased probability for moderate endoscopic inflammation (62%), while the probability for remission decreases to 6%.

**Table 5:** Predictive probabilities for the endoscopic score categories (as calculated by our Shiny web application) for preselected C-reactive protein and fecal calprotectin values.

Fecal calprotectin (mg/kg)	C-reactive protein (mg/L)					Endoscopic score
	1	5	10	20	100	
1	56% 14% 26% 4%	39% 15% 38% 8%	33% 14% 42% 11%	27% 13% 45% 15%	17% 10% 47% 26%	Remission Mild Moderate Severe
50	17% 14% 58% 11%	8% 8% 61% 23%	6% 6% 58% 30%	4% 5% 53% 38%	2% 3% 37% 58%	Remission Mild Moderate Severe
100	12% 12% 62% 14%	6% 6% 60% 28%	4% 4% 55% 37%	3% 3% 48% 46%	2% 2% 31% 65%	Remission Mild Moderate Severe
200	9% 9% 64% 18%	4% 4% 57% 35%	3% 3% 50% 44%	2% 2% 42% 54%	1% 1% 25% 73%	Remission Mild Moderate Severe
500	6% 6% 62% 26%	2% 3% 49% 46%	2% 2% 40% 56%	1% 2% 32% 65%	0% 1% 18% 81%	Remission Mild Moderate Severe

The color of the background indicates the category with the highest probability (remission: blue, moderate: orange, severe: red).

## 4. Discussion

Induction and maintenance of clinical remission, characterized by the absence of mucosal damage and inflammation, is one main focus of IBD treatment (27). Therefore, this study presented a combination of statistical methods (a Bayesian ordinal regression model and a PPFS) which may be used to develop an easy-to-use DAI for pediatric CD based on noninvasive and standard laboratory parameters as candidate predictors and endoscopic inflammation as the outcome. The improvement of proxies of endoscopic mucosal healing is increasingly important for the management of CD patients and for the assessment of new treatments, e.g., in clinical trials. Especially the retrospective nature of our study, the rather small amount of data, and the lack of an external validation cohort should be taken into account for the interpretation of our results and their discussion hereafter. In particular, our study cannot go beyond a proof of concept and does not try to establish a novel DAI for pediatric CD. Instead, our work has a methodological focus and could be used as a kind of “recipe” for the statistical part of a larger and prospective future study.

Our analysis revealed that the two routinely measured laboratory parameters CRP and FC are sufficient for predicting endoscopic inflammation. The inclusion of other parameters did not improve the predictive performance. CRP is an acute-phase protein primarily synthesized in the liver (28) and commonly used to monitor inflammatory states (29). FC is a marker that is more specific for intestinal infection/inflammation (29–31). Both parameters (CRP and FC) are not exclusive markers for CD or IBD in general (32). In particular, elevated CRP levels might be related to other inflammatory disorders than CD or individual factors such as age, sex, and body mass index (33). For example, infectious gastroenteritis or severe viral or bacterial infections may cause CRP levels to be elevated, masking CD-associated CRP elevations. If there is evidence of such an infection, it is preferable to wait until the acute infection has cleared to assess the endoscopic inflammation. In case a future DAI based on our statistical approach (and on data from a prospective study) is used to predict endoscopic inflammation, medical judgment should still be made regarding the presence of other acute inflammatory diseases to avoid false positive findings.

In contrast to CRP, it is assumed that FC reflects the degree of intestinal inflammation more likely than CRP (29–31) and correlates well with endoscopic activity in CD (34, 35). In the present study, endoscopic disease activity was associated with increased CRP and FC levels, confirming previously described findings (28, 35). However, we detected low CRP levels of ≤1 mg/L in some patients with a corresponding severe endoscopic inflammation. The attenuated CRP response might be related to potential genetic polymorphisms in the CRP gene or other interindividual factors resulting in insufficient CRP production, observed in 20%–25% of CD patients (32, 33). Moreover, the FC levels in our data displayed a high variance in children diagnosed with endoscopic remission. This might be related to the circumstance that FC levels were measured in a period of ≤30 / ≥0 days before endoscopy. A measurement immediately before endoscopy might improve the certainty and should be considered in further prospective studies.

In our study, most laboratories had a lower limit of CRP detection of 1 mg/L or 0.6 mg/L, hence the clustering of several observations at these values (see Figure 3A, for example). We are aware that there are sophisticated statistical methods for dealing with censored predictors, but such refinement would have been out of the scope of our proof-of-concept study, so we leave it for future research.

The selection of CRP and FC in our proof-of-concept study confirms the results of a prospective study in pediatric CD (6), revealing CRP and FC to be the (currently) best noninvasive biomarkers for endoscopic disease severity, while PCDAI was unreliable. A final assessment of whether CRP and FC alone are sufficient as predictors for endoscopic inflammation or whether other predictors (possibly even a completely different predictor combination lacking CRP and/or FC—although this is unlikely, given existing studies) improve the model’s predictive performance can only be made once our statistical approach has been applied to data from a larger and prospective study involving multiple cohorts.

Comparing the predictive performance between our provisional DAI (the SESM) and previously described DAIs indicated a superiority of the SESM. The SESM even had a slightly better performance than the MINI. The MINI (11), developed in 2019, identified FC, CRP, ESR, and stool frequency/consistency as predictive markers for endoscopic inflammation. Although both serum markers are preferred, the calculation can also be performed with CRP or ESR alone. In our study, data on ESR were unavailable for many visits. Therefore, the ESR could not be taken into account here, and the MINI was calculated with FC, CRP, and stool frequency.

In our analyses, the existing DAIs and our SESM show some uncertainty (across visits) regarding their predictive performance (Figure 6A), which may be related to the rather small amount of retrospectively collected data as well as to individual patient characteristics. It is probably for the same reasons that the median predictive probability for the observed endoscopic score category is often at a comparably low value of ca. 50% (Figure 6A). Therefore, applying our statistical approach in a prospective study with multiple cohorts will be necessary for validation and may avoid bias.

For Figure 6, we implicitly assumed that the continuous scores underlying the existing DAIs had linear effects on the latent predictor in their respective ordinal regression models. In the Supplementary Appendix, we provide a sensitivity analysis showing that our results would not have changed much when allowing these effects to be nonlinear. If a future study compares the predictive performance of the DAIs in the same way as we do, we recommend performing such a sensitivity analysis as well.

When comparing our SESM to the existing DAIs, it has to be kept in mind that our SESM gives probabilistic predictions by construction whereas we had to resort to auxiliary regression models for the existing DAIs. Hence, our prediction approach is considerably different from that of the existing DAIs. We mention this not only as a caveat of the comparison, but also to emphasize that the SESM is already more desirable from a conceptual perspective because it propagates uncertainty in a principled way.

In conclusion, it should be noted that the integration of DAIs in clinical practice is still a challenge. Currently, available DAIs for CD may be time-consuming in everyday practice as the collection and scoring of various data are needed. Our work serves as a proof of concept, showing that the statistical methods applied here can identify biomarkers relevant for predicting a clinical outcome such as endoscopic inflammation. Afterwards, for a new patient or visit, the values for the identified biomarkers may be entered into a web application to calculate the activity “index”, which here consists of four probabilities (one for each outcome category). Thus, the need for manual scoring is eliminated, which allows for an easy application in everyday clinical practice.

Applying our statistical methods to data from a large prospective study, including multiple cohorts, should make our predictions more reliable. In such a future application of our proposed methodology, we also recommend to train all DAIs on a common dataset (see the Supplementary Appendix), to evaluate them using an external validation cohort, and to perform a prior sensitivity analysis for the suggested Bayesian reference model. We emphasize that such a future study should not restrict the set of candidate predictors compared to our study (e.g., by excluding clinical characteristics): Even though most of our candidate predictors did not make it into the selected submodel, it is still important to allow for their potential selection based on new data. Of course, other (new) candidate predictors may always be considered additionally.

Finally, we note that the statistical approach applied here can be adapted easily to the determination of the best predictors for any clinical (or even non-clinical) outcome. The predictor ranking based on the projection-predictive feature selection keeps the number of necessary predictors at a minimum without compromising predictive performance compared to the reference model. In the context of pediatric CD, this might be an advantage for the integration of new DAIs into clinical practice in order to facilitate the clinical management of IBD. In particular, telemedicine (which might become increasingly relevant in the future) could benefit from noninvasive scores that allow an assessment based on common laboratory parameters, as physical examination is not possible.

## Data Availability

The datasets and source code for this study can be found in the Open Science Framework (OSF) at https://doi.org/10.17605/OSF.IO/EMWGP.
