# Corrigendum: Identifying predictors of clinical outcomes using the projection-predictive feature selection—a proof of concept on the example of Crohn's disease

**DOI:** 10.3389/fped.2024.1420531

**Published:** 2024-05-08

**Authors:** Elisa Wirthgen, Frank Weber, Laura Kubickova-Weber, Benjamin Schiller, Sarah Schiller, Michael Radke, Jan Däbritz

**Affiliations:** ^1^ Department of Pediatrics, Rostock University Medical Center, Rostock, Germany; ^2^ Institute for Biostatistics and Informatics in Medicine and Ageing Research, Rostock University Medical Center, Rostock, Germany; ^3^ Medical School, University of Rostock, Rostock, Germany; ^4^ Department of Pediatrics, Pediatric Gastroenterology, Rostock University Medical Center, Rostock, Germany; ^5^ Department of Pediatrics, Greifswald University Medical Center, Greifswald, Germany

**Keywords:** inflammatory bowel disease, endoscopy, calprotectin, C-reactive protein, monitoring, Bayesian, ordinal regression model, shiny application

A Corrigendum on Identifying predictors of clinical outcomes using the projection-predictive feature selection–a proof of concept on the example of Crohn’s disease By Wirthgen E, Weber F, Kubickova-Weber L, Schiller B, Schiller S, Radke M, Däbritz J. (2023) Identifying predictors of clinical outcomes using the projection-predictive feature selection-a proof of concept on the example of Crohn's disease. Front Pediatr 11:1170563. doi: 10.3389/fped.2023.1170563

In the published article, there was an error in the Supplementary Material (Data Sheet 1). The source code mentioned in the “Data Availability Statement“ has been corrected in the Open Science Framework (OSF) at https://doi.org/10.17605/OSF.IO/EMWGP. This correction of the source code affects a number mentioned in the Supplementary Material (section “2.1 Reference model”, page 6): “The refit for this single visit (see section 1.4 here in the Supplementary Appendix) was successful, yielding an estimate for the effective number of parameters of approximately 38.6, which is much smaller than the number of visits (131, after exclusion of visits with missing values) and the total number of model parameters (118), as desired.” The correct material statement appears below.

“The refit for this single visit (see section 1.4 here in the Supplementary Appendix) was successful, yielding an estimate for the effective number of parameters of approximately 38.6, which is much smaller than the number of visits (131, after exclusion of visits with missing values) and the total number of model parameters (143), as desired.”

The authors apologize for this error and state that this does not change the scientific conclusions of the article in any way. The original Supplementary Material of the article has been updated.

