# Supplementary material for: Identifying predictors of clinical outcomes using the projection-predictive feature selection—a proof of concept on the example of Crohn’s disease
Source: Front Pediatr. 2023 Jul 28;11:1170563. doi: 10.3389/fped.2023.1170563 (PMC10420065; doi:10.3389/fped.2023.1170563)
Supplement: Supplementary file 1 [file Datasheet1.pdf]

Supplementary Appendix for "Identifying predictors of clinical outcomes using the projection-predictive feature selection – a proof of concept on the example of Crohn's disease"

1 Statistical methods

1.1 Rationale

The rationale behind our statistical approach (a Bayesian ordinal regression model and a projection-predictive feature selection) is as follows:

1. Existing disease activity indices (DAIs) (1–6) largely rely on the discretization of predictors, which comes with a loss of information and precision.
2. Regression models provide a natural way for prediction (7) and can avoid the discretization of predictors.
3. Since the outcome (the endoscopic score) is ordinal, an ordinal regression model is the most appropriate one.
4. Bayesian statistics has various advantages compared to frequentist statistics, most notably a more intuitive interpretation (8–10). Thus, it makes sense to use a Bayesian ordinal regression model.
5. In a Bayesian framework, the projection-predictive feature selection (PPFS) implemented in the R (11) package projpred (12–14) is a state-of-the-art variable selection method (13–15) which is especially suitable for prediction problems like in our case. In particular, the PPFS allows for valid post-selection inference (apart from the potential—but usually small (15)—overfitting induced by the selection of the submodel size) because it does not discard the uncertainty inherent to the reference model (here the full model containing all candidate predictors) (15).

The rather complex calculation of the predictive probabilities (based on the model selected by the PPFS) is performed most conveniently by a Shiny (16) web application that users interact with *via* a simple graphical interface.

In settings with a binary outcome, a receiver operating characteristic (ROC) curve is a widely-applied and useful tool to compare different diagnostic tests (or DAIs, which may be considered as diagnostic tests). In our case, however, the outcome (the endoscopic score) has four categories, hence the need for a more general method.

1.2 Bayesian statistics

An introduction to Bayesian ordinal regression models (especially in the context of the R package brms (17–19)) is given in (20). For readers not familiar with Bayesian statistics (in general), we give a short introduction here, taken mainly from the shinybrms (21) application: The fundamental principle of Bayesian statistics is Bayes' theorem. In the context relevant for this publication, Bayes' theorem may be simplified to the statement that the joint posterior density of all parameters in the regression model is proportional to the product of their joint prior density and the likelihood. The

posterior distribution (corresponding to the posterior density) reflects the knowledge about the parameters after seeing the data. The prior distribution (corresponding to the prior density) reflects the knowledge about the parameters before having seen the data. The likelihood corresponds to the distribution of the outcome conditional on the parameters and the predictors. Thus, after having specified the likelihood and the prior (distribution), a Bayesian data analysis aims to infer the posterior (distribution) and then to perform analyses based on the posterior, e.g., by plotting marginal posterior distributions and calculating their 2.5%, 50%, and 97.5% quantiles. Inferring the posterior is the Bayesian way to estimate unknown parameters.

1.3 Projection-predictive feature selection

The projection-predictive feature selection (PPFS) implemented in the R package projpred (12–14) is a predictor selection method whose resulting models have a predictive performance close to the gold standard prediction method (Bayesian model averaging or—equivalently—a spike-and-slab prior applied to the full model) (15). In the following, we give more details on the PPFS in general as well as on its use in our proof-of-concept study. In doing so, we mention to which values the arguments of involved R functions are set, at least for the most important ones. The full R code is supplied together with our manuscript.

The PPFS requires a reference model, which should be the best model (in terms of predictive performance) one can construct (13). Our approach of constructing the reference model from the full model (i.e., including all candidate predictors) with a regularized horseshoe (RH) prior (22) is a standard approach for constructing the reference model, as previously described (13–15, 23). The RH prior is a so-called "shrinkage" prior for population-level regression coefficients (also termed "fixed" effects). The idea of the RH prior is to shrink irrelevant coefficients to zero and leave relevant coefficients as they are. We infer the global scale parameter of the RH prior from the ratio of the number of *a priori* deemed relevant regression coefficients to the number of *a priori* deemed irrelevant regression coefficients. Thereby, we set the number of *a priori* deemed relevant regression coefficients to 5, which is based on a rough guess of the total number of regression coefficients implied by the existing disease activity indices (DAIs). However, we have found our results to be largely insensitive to this number of *a priori* deemed relevant regression coefficients.

With the reference model set up, the PPFS first tries to find the smallest subset of predictors which still achieves a predictive performance as close as possible to the reference model's predictive performance. To avoid the investigation of all possible subsets of predictors (i.e., all possible submodels, which is computationally expensive), the PPFS runs a heuristic search to identify a single (best) submodel for each submodel size. For a multilevel reference model as in our case, the so-called "forward search" is the only option available in projpred. During this forward search, the reference model is "projected" onto each submodel that the search passes through (in fact, this projection is a central component of the PPFS and achieved by minimizing the discrepancy from the reference model's predictive distribution to the respective submodel's predictive distribution). For the "cumulative" outcome family we are using here, we apply the latent projection (24) as implemented in projpred. For post-projection analyses, we rely on projpred's default of so-called "response-scale analyses" which means that internally, the ordinal intercepts (the latent category thresholds) from the reference model are employed to turn a latent Gaussian submodel back into an ordinal regression model. To save computation time, we stop the forward search at submodel size 3 (this is sufficient here to see that size 2 should be chosen, see Figure 2 of the main article). For the number of clusters (of posterior draws) employed during that search, we use the default value of 20. We also tried a high

value of 800 clusters (results not shown), but the full-data predictor ranking stayed the same and the fold-wise predictor rankings essentially, too (there were only two CV folds with very minor differences in the predictor rankings). Thus, we picked the default value of 20 clusters which is computationally easier to handle. After the search part, the PPFS consists of a performance evaluation part, yielding a plot of a user-specified predictive performance measure in dependence of the submodel size. As predictive performance measure, we choose the mean log predictive density (MLPD) because it is well-established (13, 25) and because exponentiating it to the base of the natural logarithm gives the geometric mean predictive density (GMPD) as a closely related predictive performance measure which has an intuitive interpretation in our case: Here, the GMPD is the geometric mean of the predictive probabilities at the observed outcome categories (and hence restricted to the interval [0, 1]). For the creation of the predictive performance plot that is used for deciding a submodel size, the projpred package projects the reference model onto the best submodel of each size again. In these re-projections, we use 2666 thinned posterior draws (2666 is calculated as 8000 / 3, rounded down to the nearest integer; 8000 is the number of post-warmup posterior draws for the reference model, see section 1.4 here in the Supplementary Appendix). We cannot use the full 8000 posterior draws but only approximately every third of them due to computational constraints. However, since results were only slightly affected by our change from the default of 400 thinned draws (not shown here) to 2666 thinned draws, we think that switching to the full 8000 draws would have had only a very minor impact on the results. By inspecting the predictive performance plot created from the PPFS's performance evaluation results, the user then has to select a submodel size. Here, we choose that submodel size from which on the predictive performance measure does not improve anymore.

With a chosen submodel size, the selected submodel is given by the (best) submodel corresponding to that chosen submodel size. The reference model is then projected once again onto the selected submodel. Here, we perform this final projection using the "draw-by-draw" method (13). These final projection results are used for our Shiny web application as well as for Figure 5 of the main article.

For the PPFS, it is recommended to perform a cross-validation (CV) around the heuristic search and the performance evaluation (13, 15). Here, we perform a 25-fold CV.

Furthermore, we guard against the possibility of an illegitimate advantage of our approach (which considers group-level effects for the patient identifiers (patient IDs) as a candidate predictor) compared to the existing DAIs (which do not use group-level effects, see section 1.5 here in the Supplementary Appendix) by treating the original patient IDs as new patient IDs during predictions. This is achieved by setting `projpred.mlvl_pred_new` (a global R option used by the projpred package) to `TRUE`. This choice also reflects the later prediction task in clinical practice: In the Shiny application that we propose, all user input is treated as that for a new patient because the purpose of the Shiny application is to generalize prediction beyond the dataset used for training the model.

The reason why we subject the IBD center as well as the group-level effects for the patient IDs to variable selection is simply a technical one: Within projpred, it is currently a lot easier (and—in some regard—more efficient in terms of memory used) to consider all predictors from the reference model as candidate predictors than to exclude some. Retrospectively, our approach can be justified by the fact that neither of these two terms has been among the two most relevant terms (neither in the full-data nor in the fold-wise searches; fold-wise predictor rankings not shown here for the sake of brevity). If either of these two terms had been among the two most relevant, then it would have made sense to run a final variable selection with these two terms excluded from the set of candidate

predictors. Nonetheless, first running the “simpler” variable selection (with these two terms considered as candidate predictors) can give helpful insight: If they had been among the two most relevant terms, this would have meant that they are relevant for prediction, which would have revealed some important peculiarity of the data.

1.4 Reference model fit and analysis

For fitting our reference model, we increase the number of Markov chain Monte Carlo (MCMC) iterations per Markov chain from the default (2000) to 4000. Given that half of this number is for warmup (by default) and given the default of 4 Markov chains, this yields a total of 8000 post-warmup posterior draws used for inference here. Furthermore, we set the target Metropolis acceptance rate to 0.99 (the default in the R package rstanarm (26), for example, is 0.95 but we had to increase it a little further to aim for smaller step sizes of the underlying special MCMC sampler and hence to eliminate spurious divergences) and the maximum tree depth to 15 (which is the default for most rstanarm models, for example).

As part of a Bayesian modeling workflow (27), it is recommended to perform posterior predictive checks (PPCs): A good model should be able to generate outcome values close to the observed ones when giving the observed data to the model and letting the model learn from it. Here, we do not present PPCs for the reference model since the reference model should have a PPC performance at least as good as the selected submodel. We were able to confirm this in our case (results not shown). Furthermore, Figure 6 of the main article includes the predictive performance of the selected submodel and thus can also be interpreted as a conservative out-of-sample PPC for the reference model.

As another step to ensure that we have a reasonable reference model, we use the R package loo (28) to estimate the Pareto k-values and the effective number of parameters from a Pareto-smoothed importance sampling leave-one-out cross-validation (PSIS-LOO CV) (29, 30):

1. For each observation (visit), we obtain a single estimated Pareto k-value. High Pareto k-values (> 0.7) indicate highly influential observations (29, 30), which is usually undesired and often requires adjustments to the model. Even more importantly, they also indicate that the corresponding PSIS-LOO CV weights (as well as all downstream quantities based upon them, including the effective number of parameters from the next step) may be unreliable.
2. After inspecting the Pareto k-values from the original PSIS-LOO CV, we update the PSIS-LOO CV results for visits with a Pareto k-value > 0.7 by refitting with each of these observations left out, as implemented in the brms package. Then, after these updates, we obtain a single effective number of parameters for the whole model. We wish to have an effective number of parameters smaller than the number of visits and smaller than the total number of parameters in the model (31).

Since the primary purpose of our regression model is prediction, we do not visualize the marginals of the selected submodel’s projected posterior directly, but we create conditional-effects plots (Figure 5 of the main article) whose interpretation is explained in the respective figure caption.

1.5 Comparison of the selected submodel to existing pediatric Crohn's disease activity indices

Missing values in our dataset caused different numbers of usable visits for the different DAIs when performing a complete-case analysis for each DAI. In principle, it would have been desirable to train the DAIs and cross-validate their predictive performance on a common dataset (the largest possible one), but reducing our proof-of-concept dataset to the largest possible common dataset across all DAIs would have led to only 46 visits. Thus, the comparison of the DAIs presented in Figure 6 of the main article takes the maximum possible number of visits for a separate complete-case analysis of each DAI (which results in datasets of differing sizes for the different DAIs, as indicated by "N" in Figure 6). A future validation study of our proof-of-concept analysis presented here should conduct the comparison of all DAIs on a common dataset.

The comparison itself takes place *via* cross-validated predictive probabilities (CV-PPs; termed "predictive probabilities for the observed endoscopic inflammation" in the main article): First, for each existing DAI, we fit one Bayesian ordinal regression model similar to the reference model described in section 2.4 of the main article (and in sections 1.3 and 1.4 here in the Supplementary Appendix), but with the respective DAI (before any categorization of its score value, i.e., using its underlying continuous score) as the only predictor. For the regression coefficient of this single predictor, we use brms's default (flat) prior. (In principle, a flat prior for regression coefficients is not recommended due to the risk of overfitting, but in our case, a more restrictive prior could have meant a systematic disadvantage for the existing DAIs, and thus an advantage for our selected submodel. In this regard, the flat prior is a conservative choice with respect to the comparison with our selected submodel.) Then, for a given DAI (either existing or the selected submodel) and a given visit $i \in \{1, \dots, N\}$ (with N denoting the number of visits), the CV-PP is the probability given to the actually observed outcome category of visit i by the DAI's regression model when leaving the CV fold containing visit i out of the model building process. Thus, higher CV-PPs are better than smaller ones. For the existing DAIs, we use a 25-fold CV, as in the PPFS yielding the selected submodel. Finally, the CV-PPs are illustrated using separate boxplots (one per DAI) with the individual values (those of which the boxplots consist) overlaid as jittered points. Furthermore, we use such jittered boxplots also for the CV-PP differences, comparing the selected submodel *vs.* each existing DAI so that a CV-PP difference greater than zero speaks in favor of the selected submodel.

For each of the existing DAIs, this CV-PP approach implicitly assumes that its underlying continuous score has a linear effect on the latent predictor of its ordinal regression model. In section 2.2, we provide a sensitivity analysis showing that our results would not have changed much when allowing these effects to be nonlinear (using brms's built-in support for smooth terms; details are provided in our publicly available R code).

1.6 Other R packages

Other R packages used here are data.table (32), readxl (33), ggplot2 (34, 35), posterior (36), and bayesplot (37, 38). For brms, we use the cmdstanr (39) backend.

2 Results

2.1 Reference model

In the reference model, we observed only a single visit with a high Pareto k-value (> 0.7). Given such a small number of highly influential observations, we consider this model to be adequate in this

regard. The refit for this single visit (see section 1.4 here in the Supplementary Appendix) was successful, yielding an estimate for the effective number of parameters of approximately 38.6, which is much smaller than the number of visits (131, after exclusion of visits with missing values) and the total number of model parameters (143), as desired.

2.2 Sensitivity analysis for nonlinear effects of the existing DAIs

Figure A1 replicates Figure 6 from the main article, but allowing for nonlinear effects in the existing DAIs' ordinal regression models. As can be seen by comparing Figure A1A to Figure 6A, this extra flexibility mostly leads to very similar CV-PP values, a noticeable exception being the MINI for which the median CV-PP increases from ca. 45% to ca. 50%. Interestingly, however, comparing Figure A1B to Figure 6B reveals that even for the MINI, this extra flexibility does not change the CV-PP difference to the selected submodel considerably (for the MINI, the median CV-PP difference even increases when allowing for nonlinear effects, hence speaking more strongly in favor of the selected submodel).

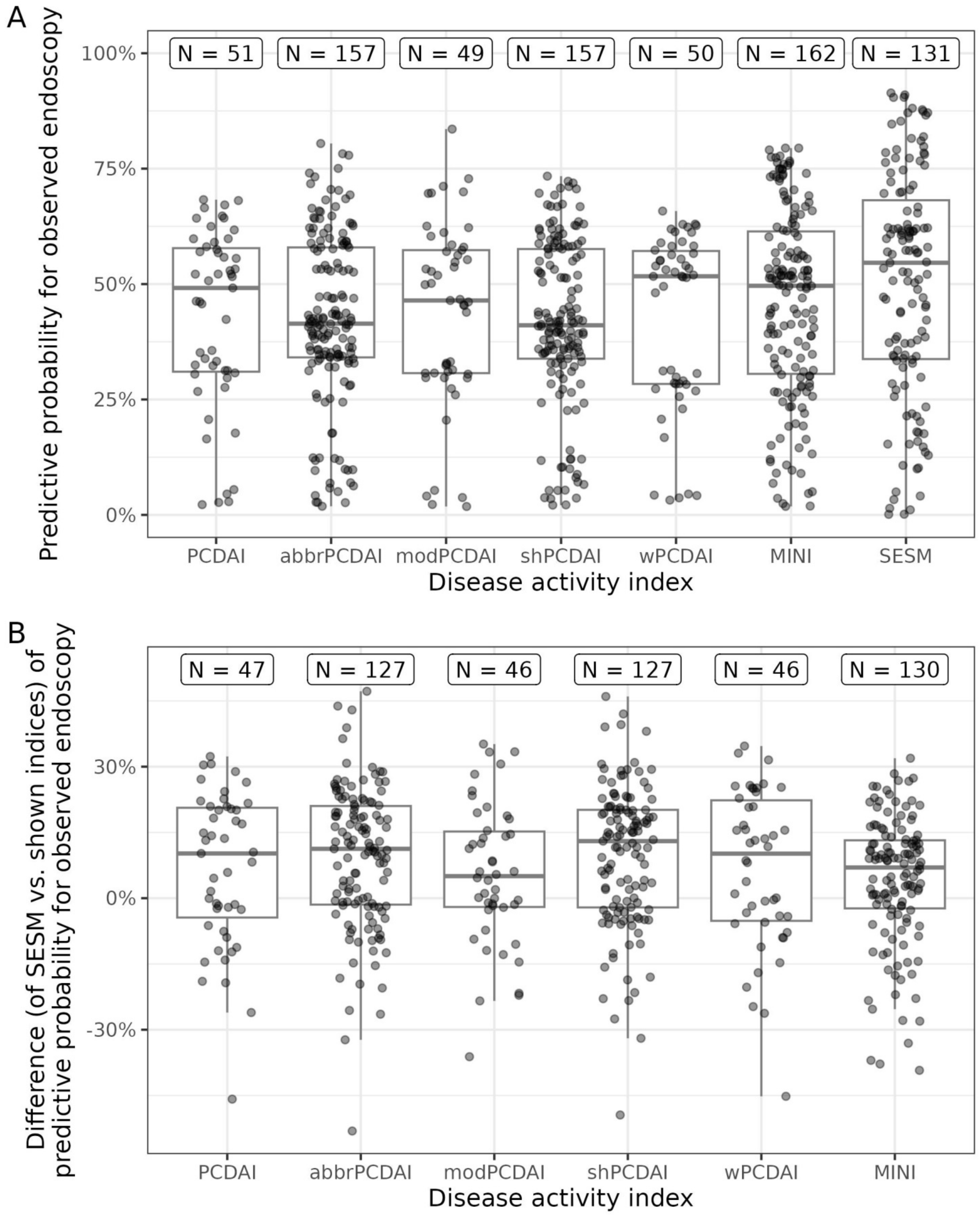

Figure A1. The same as Figure 1 from the main article, but allowing for nonlinear effects in the existing DAIs' ordinal regression models.

3 REFERENCES

1. Hyams JS, Ferry GD, Mandel FS, Gryboski JD, Kibort PM, Kirschner BS, et al. Development and validation of a pediatric Crohn's disease activity index. *J Pediatr Gastroenterol Nutr* (1991) **12**:439–47.

2. Shepanski MA, Markowitz JE, Mamula P, Hurd LB, Baldassano RN. Is an abbreviated Pediatric Crohn's Disease Activity Index better than the original? *J Pediatr Gastroenterol Nutr* (2004) **39**:68–72.

3. Leach ST, Nahidi L, Tilakaratne S, Day AS, Lemberg DA. Development and assessment of a modified Pediatric Crohn Disease Activity Index. *J Pediatr Gastroenterol Nutr* (2010) **51**:232–6. doi:10.1097/MPG.0b013e3181d13609

4. Kappelman MD, Crandall WV, Colletti RB, Goudie A, Leibowitz IH, Duffy L, et al. Short pediatric Crohn's disease activity index for quality improvement and observational research. *Inflamm Bowel Dis* (2011) **17**:112–7. doi:10.1002/ibd.21452

5. Turner D, Griffiths AM, Walters TD, Seah T, Markowitz J, Pfefferkorn M, et al. Mathematical weighting of the Pediatric Crohn's Disease Activity Index (PCDAI) and comparison with its other short versions. *Inflamm Bowel Dis* (2011) **18**:55–62. doi:10.1002/ibd.21649

6. Cozijnsen MA, Ben Shoham A, Kang B, Choe BH, Choe YH, Jongsma MM, et al. Development and validation of the Mucosal Inflammation Noninvasive Index for pediatric Crohn's disease. *Clin Gastroenterol Hepatol* (2020) **18**:133-140.e1. doi:10.1016/j.cgh.2019.04.012

7. Gelman A, Hill J, Vehtari A. Regression and Other Stories. 1st ed. Cambridge, UK: Cambridge University Press (2020).

8. Gelman A, Carlin JB, Stern HS, Dunson DB, Vehtari A, Rubin DB. Bayesian Data Analysis. 3rd ed. Boca Raton, FL, USA: CRC Press (2014).

9. StataCorp. Stata Bayesian Analysis Reference Manual. 16th ed. College Station, TX, USA: Stata Press (2019).

10. Weber F, Ickstadt K, Glass Ä. shinybrms: Fitting Bayesian regression models using a graphical user interface for the R package brms. *R J* (2022) **14**:96–120. doi:10.32614/RJ-2022-027

11. R Core Team. R: A Language and Environment for Statistical Computing [computer program on the Internet]. Version 4.3.0. Vienna, Austria: R Foundation for Statistical Computing (2023). Available at: https://www.R-project.org/.

12. Piironen J, Paasiniemi M, Catalina A, Weber F, Vehtari A. projpred: Projection Predictive Feature Selection [computer program on the Internet]. R package, version 2.6.0.9000 (from https://github.com/stan-dev/projpred/tree/190a07d4e90d4d2bdc9d8b03a46fcd34a8b05e71) (2023). Available at: https://mc-stan.org/projpred/.

13. Piironen J, Paasiniemi M, Vehtari A. Projective inference in high-dimensional problems: prediction and feature selection. *Electron J Stat* (2020) **14**:2155–97. doi:10.1214/20-EJS1711

14. Catalina A, Bürkner P-C, Vehtari A. Projection predictive inference for generalized linear and additive multilevel models. arXiv [Preprint] (2020). Available at: https://arxiv.org/abs/2010.06994v1 (Accessed November 03, 2020).

15. Piironen J, Vehtari A. Comparison of Bayesian predictive methods for model selection. *Stat Comput* (2017) **27**:711–35. doi:10.1007/s11222-016-9649-y

16. Chang W, Cheng J, Allaire JJ, Sievert C, Schloerke B, Xie Y, et al. shiny: Web Application Framework for R [computer program on the Internet]. R package, version 1.7.4 (2022). Available at: https://CRAN.R-project.org/package=shiny.

17. Bürkner P-C. brms: Bayesian Regression Models using 'Stan' [computer program on the Internet]. R package, version 2.19.0 (2023). Available at: https://paul-buerkner.github.io/brms/.

18. Bürkner P-C. brms: an R package for Bayesian multilevel models using Stan. *J Stat Softw* (2017) **80**:1–28. doi:10.18637/jss.v080.i01

19. Bürkner P-C. Advanced Bayesian multilevel modeling with the R package brms. *R J* (2018) **10**:395–411. doi:10.32614/RJ-2018-017

20. Bürkner P-C, Vuorre M. Ordinal regression models in psychology: a tutorial. *Adv Methods Pract Psychol Sci* (2019) **2**:77–101. doi:10.1177/2515245918823199

21. Weber F. shinybrms: Graphical User Interface ('shiny' App) for 'brms' [computer program on the Internet]. R package, version 1.8.0 (2022). Available at: https://fweber144.github.io/shinybrms/.

22. Piironen J, Vehtari A. Sparsity information and regularization in the horseshoe and other shrinkage priors. *Electron J Stat* (2017) **11**:5018–51. doi:10.1214/17-EJS1337SI

23. Pavone F, Piironen J, Bürkner P-C, Vehtari A. Using reference models in variable selection. *Comput Stat* (2022). doi:10.1007/s00180-022-01231-6

24. Catalina A, Bürkner P, Vehtari A. Latent space projection predictive inference. arXiv [Preprint] (2021). Available at: https://arxiv.org/abs/2109.04702v1 (Accessed September 30, 2021).

25. Vehtari A, Ojanen J. A survey of Bayesian predictive methods for model assessment, selection and comparison. *Stat Surv* (2012) **6**:142–228. doi:10.1214/12-SS102

26. Goodrich B, Gabry J, Ali I, Brilleman S. rstanarm: Bayesian Applied Regression Modeling via Stan [computer program on the Internet]. R package, version 2.21.4 (2023). Available at: https://mc-stan.org/rstanarm/.

27. Gelman A, Vehtari A, Simpson D, Margossian CC, Carpenter B, Yao Y, et al. Bayesian workflow. arXiv [Preprint] (2020). Available at: https://arxiv.org/abs/2011.01808v1 (Accessed November 14, 2020).

28. Vehtari A, Gabry J, Magnusson M, Yao Y, Bürkner P-C, Paananen T, et al. loo: Efficient Leave-One-Out Cross-Validation and WAIC for Bayesian Models [computer program on the Internet]. R package, version 2.6.0 (2023). Available at: https://mc-stan.org/loo/.

29. Vehtari A, Gelman A, Gabry J. Practical Bayesian model evaluation using leave-one-out cross-validation and WAIC. *Stat Comput* (2017) **27**:1413–32. doi:10.1007/s11222-016-9696-4

30. Vehtari A, Simpson D, Gelman A, Yao Y, Gabry J. Pareto smoothed importance sampling. arXiv [Preprint] (2022). Available at: https://arxiv.org/abs/1507.02646v8 (Accessed February 17, 2023).

31. Vehtari A, Gabry J, Magnusson M, Yao Y, Bürkner P-C, Paananen T, et al. LOO package glossary (2022). https://mc-stan.org/loo/reference/loo-glossary.html [Accessed May 27, 2022].

32. Dowle M, Srinivasan A. data.table: Extension of ‘data.frame‘ [computer program on the Internet]. R package, version 1.14.8 (2023). Available at: https://CRAN.R-project.org/package=data.table.

33. Wickham H, Bryan J. readxl: Read Excel Files [computer program on the Internet]. R package, version 1.4.2 (2023). Available at: https://CRAN.R-project.org/package=readxl.

34. Wickham H. ggplot2: Create Elegant Data Visualisations Using the Grammar of Graphics [computer program on the Internet]. R package, version 3.4.2 (2023). Available at: https://ggplot2.tidyverse.org.

35. Wickham H. ggplot2: Elegant Graphics for Data Analysis. 2nd ed. New York: Springer (2016). 260 p.

36. Bürkner P-C, Gabry J, Kay M, Vehtari A, Magnusson M, Češnovar R, et al. posterior: Tools for Working with Posterior Distributions [computer program on the Internet]. R package, version 1.4.1 (2023). Available at: https://mc-stan.org/posterior/.

37. Gabry J, Mahr T. bayesplot: Plotting for Bayesian Models [computer program on the Internet]. R package, version 1.10.0 (2022). Available at: https://mc-stan.org/bayesplot/.

38. Gabry J, Simpson D, Vehtari A, Betancourt M, Gelman A. Visualization in Bayesian workflow. *J R Stat Soc Ser A Stat Soc* (2019) **182**:389–402. doi:10.1111/rssa.12378

39. Gabry J, Češnovar R. cmdstanr: R Interface to ’CmdStan’ [computer program on the Internet]. R package, version 0.5.3 (2022). Available at: https://mc-stan.org/cmdstanr/.
